# Therapy response of glucocorticoid-refractory acute GVHD of the lower intestinal tract

**DOI:** 10.1038/s41409-022-01741-3

**Published:** 2022-06-29

**Authors:** Francesca Biavasco, Gabriele Ihorst, Ralph Wäsch, Claudia Wehr, Hartmut Bertz, Jürgen Finke, Robert Zeiser

**Affiliations:** 1 Department of Medicine I, Medical Center—University of Freiburg, Faculty of Medicine, Albert Ludwigs University (ALU), Freiburg, Germany; 2 Clinical Trials Center Freiburg, Faculty of Medicine, Albert Ludwigs University (ALU), Freiburg, Germany; 3 German Cancer Consortium (DKTK), Freiburg, Germany; 4 Signalling Research Centres BIOSS and CIBSS—Centre for Integrative Biological Signalling Studies, University of Freiburg, Freiburg, Germany; 5 German Cancer Research Center (DKFZ), Heidelberg, Germany

**Keywords:** Clinical trials, Translational research

## Abstract

Acute graft-versus-host disease (aGVHD) is a major life-threatening complication of allogeneic hematopoietic cell transplantation. While most studies report therapy-response of aGVHD including a cumulative grade of skin, liver and intestinal tract manifestations, there is a lack of information specifically on lower gastrointestinal tract aGVHD (GI-GVHD) therapy-response, which is highly relevant in light of novel therapies that target intestinal regeneration such as IL-22, R-spondin or GLP-2. Here we retrospectively analyzed patients who developed GI-GVHD over a 6-year period. A total of 144 patients developed GI-GVHD and 82 (57%) were resistant to glucocorticoid-therapy (SR). The most commonly used second-line therapy was ruxolitinib (74%). Overall and complete response (CR) to ruxolitinib on day 28 were 44.5% and 13%, respectively. SR-GVHD patients experienced a lower 5-year overall survival (OS) (34.8 vs 53.3%, *p* = 0.0014) and higher incidence of 12-months non-relapse-mortality (39.2 vs 14.3%, *p* = 0.016) compared to glucocorticoid-sensitive patients. SR-GI-GVHD patients, that achieved a CR on day 28 after ruxolitinib start, experienced a higher OS compared to non-CR patients (*p* = 0.04). These findings indicate that therapy response of SR-GI-GVHD to different immunosuppressive approaches is still low, and that novel therapies specifically aiming at enhanced intestinal regeneration should be tested in clinical trials.

## Introduction

Allogeneic hematopoietic cell transplantation (allo-HCT) represents the only potentially curative therapy for the majority of high-risk hematological malignancies [1] and the number of allo-HCTs performed worldwide is increasing [2, 3]. Acute graft-versus-host disease (GVHD) is a major complication of allo-HCT [4, 5] affecting 20–80% of the patients [6–8] and accounting for 15–30% of the mortality rate [9, 10]. According to the EBMT guidelines the first-line treatment of acute GVHD grade 2–4 is systemic glucocorticoid [11]. However, ~60–70% of patients with severe GVHD and around 40% of patients with mild-moderate GVHD show resistance to initial therapy (glucocorticoid refractory GVHD; SR-GVHD) or inability to taper glucocorticoids [12, 13]. Outcome of SR-GVHD is dismal, reaching up to 80% mortality at 1 year after diagnosis [6, 14]. The treatment of GVHD affecting the lower gastrointestinal tract (GI-GVHD) is particularly challenging, due to the fluid- and electrolyte loss caused by secretory diarrhea, the risk of severe bleeding, especially if concomitant thrombocytopenia is present, and the frequent overlap with bacterial and viral infection in highly susceptible immunocompromised patients. In line with that, GI-GVHD is the GVHD subtype connected to the highest non-relapse mortality after allo-HCT [15, 16]. Moreover, gastrointestinal involvement has been reported to be one of the major risk factors for glucocorticoid resistance (HR = 5.9, *p* < 0.01, [17]). Many different immunosuppressive agents are available as second-line therapy (SLT) alone or in combination; no clear superiority of one agent over the others has been shown and often only short-term control of the disease can be achieved [6, 18, 19]. Recently, a multicenter, randomized, phase III clinical trial showed a significant improvement in overall response (OR) rate, duration of response and failure-free survival by using the JAK1/2 inhibitor ruxolitinib as SLT for acute SR-GVHD compared to control therapy of investigator´s choice from a list of nine commonly used SLT options [20]. Comparable results were reported in several real-world reports on ruxolitinib [21–23]. In recent years several approaches to improve the outcome of intestinal GVHD were tested in mice. Treatment with interleukin-22 (IL-22) [24, 25], R-spondin [26, 27] and glucagon like peptide-2 (GLP-2) [28] led to improved intestinal regeneration and reduced GVHD related mortality in mice. To test these approaches in the clinic it will be important to assess selectively the therapy response of lower intestinal tract aGVHD in a real-world setting in the era of ruxolitinib. Previous reports on GI-GVHD therapy response date back to the time before ruxolitinib was used for SR-GVHD [29, 30]. To address that, we report here incidence, therapy response and outcome of patients developing lower intestinal tract acute GVHD at the Medical Center of the University of Freiburg (MCUF) over a time period of 6 years.

## Methods

### Patients and data collection

The present analysis is a cross-sectional single centre cohort analysis, analyzing adult patients who developed acute gastrointestinal GVHD at any time point after receiving allo-HCT in the period between 14 January 2015 and 16 March 2021 at the MCUF. To provide a comprehensive overview of all patients with GI involvement, patients were included in this analysis even if GI involvement appeared after application of systemic glucocorticoid therapy due to other organs involvement (8 patients). Treatments received by these patients are described in Supplementary Table 2. GVHD was graded as previously described [31]. In total 144 patients meeting the criteria were enrolled. The analysis was conducted in accordance with the tenets of the Declaration of Helsinki and all patients gave informed consent for data collection and analysis. Ethic committee Freiburg approved the retrospective analysis of patient data (approval number 547/17). Laboratory values during the disease course and clinical outcomes were collected from medical records with data cut-off at 30 June 2021.

### Endpoints and assessment

The present analysis is focused on SR patients, defined by physician as patients with GI-GVHD progression or no improvement after standard-dose systemic glucocorticoid therapy, as recommended by EBMT Guidelines [11, 14]. In total 82 patients had SR-GVHD. SR patients were retrospectively divided in patients who progressed after at least 3 days of glucocorticoid therapy (*n* = 29), patients who did not improve after at least 7 days of glucocorticoid therapy (*n* = 37) and patients who failed to taper glucocorticoid therapy without GVHD flare (*n* = 14) (Supplementary Table 4). From two patients the definition of SR was not available. Steroid dose at GVHD onset and at begin of SLT are reported in Supplementary Table 4. Endpoints of the analysis include OR to therapy, defined as the proportion of patients who experienced a gastrointestinal complete response (CR) (complete resolution of all signs and symptoms of acute GI GVHD without administration of additional systemic therapies for GVHD flair or other organs involvement with exception of calcineurin inhibitors and tapered glucocorticoid therapy with prednisone 5 mg or less), or gastrointestinal partial response (PR) (improvement of at least two point on the basis of organ assessment without administration of additional systemic therapies for GVHD flair or other organs involvement with exception of calcineurin inhibitors and tapered glucocorticoid therapy with prednisone 5 mg or less); time to first response (time from therapy begin to time point of first assessment of PR or better); duration of response (time from first response to acute GI-GVHD progression or introduction of additional systemic therapy for acute GI-GVHD, censored at last follow-up; competing events were the evolution into chronic GVHD or death without progression of acute GI-GVHD); overall survival (OS) (time from acute GI-GVHD onset to death of any cause, censored at last follow-up); non-relapse mortality (time from acute GI-GVHD onset to death not preceded by relapse/progression of primary cancer, censored at last follow-up; competing event was relapse/progression of primary cancer) and relapse incidence (time from GI-GVHD onset to occurrence of relapse/progression of primary cancer, censored at last follow-up; competing event was death before occurrence of relapse/progression of primary cancer). Response to therapy was evaluated at day 28 after initiation of SLT, at day 56 after initiation of SLT and the timepoint of best achieved response during follow up time. Patients were excluded from the response analysis if two or more agents were simultaneously started. Patients who experienced relapse/progression of primary cancer before onset of GI-GVHD were excluded from analysis of non-relapse mortality and relapse incidence.

### Statistical analysis

Data were analyzed using GraphPad Software (Version 5.03, December 10, 2009) and SAS V9.4 (SAS Institute Inc., Cary, NC, USA). The investigator was not blinded to the group allocation during analysis of data. For group comparisons log-rank tests were used for time-to-event variables, Gray tests for time to event variables with competing risks. Normal distribution of the sample was analyzed with D’Agostino & Pearson omnibus normality test. OS rates were estimated and displayed using the Kaplan–Meier method. For OS and NRM after SLT a landmark-analysis was used, eliminating from the analysis all patients who did not reach day 28. In competing risks analyses, cumulative incidence rates were estimated and displayed. Two-tailed *p* value are reported and a *p* value < 0.05 was defined as statistically significant.

## Results

### Demographic, transplant-related and GVHD characteristics

Between January 2015 and April 2021, 144 adult patients developed acute GI-GVHD after undergoing allo-HCT at MCUF and were included in this analysis. Demographic and transplantation-related characteristics are represented in Table 1. The median age was 59 years (range 18–77) and male patients were slightly predominant (62.5%). The most common indication for allo-HCT was acute myeloid leukemia, representing 63% of the patients, followed by other myeloid malignancies (31%), non-Hodgkin lymphoma (23%), acute lymphoblastic leukemia (12%), multiple myeloma (7%), Hodgkin lymphoma (3%) and primary immunodeficiency (3%). Most of the patients (94%) developed GI-GVHD after the first allo-HCT and 9 patients (6%) were analyzed after a second allo-HCT. Similarly to the CIBMTR annual record [3], the most common donor type in our cohort was a matched unrelated donor (MUD), accounting for 45.1% of patients, followed by matched related donor (MRD) (25.7%) and by mismatched unrelated donor (MMUD) with 19.9%. Haploidentical transplantation was performed in 9.7% of patients. GVHD characteristics and organ involvement are listed in Table 2.

**Table 1:** Clinical and transplant-related characteristics.

Age at allo-Tx	Median (range)
	59 (18–77)
Gender	n (%)
Female	54 (37.5)
Male	90 (62.5)
Diagnosis	
AML	63 (44)
MDS, MPN, MDS/MPN overlap	31 (22)
NHL	23 (16)
ALL	12 (8)
MM	7 (5)
HL	3 (2)
Immunodeficiency	3 (2)
Other^a^	2 (1)
HCT number	
First HCT	135 (94)
Second HCT	9 (6)
Conditioning regimen	
MAC	124 (86)
RIC	20 (14)
Donor type	
MRD	37 (25.7)
Haploidentical	14 (9.7)
MUD	65 (45.1)
MMUD	28 (19.4)
Donor mismatch	
9–10/10	130 (90)
≤8/10	14 (10)
Gender mismatch	69 (48)
Graft source	
PBSC	143 (99)
BM	1 (1)
GvHD prophylaxis	
CSA/MMF/ATG20–30mg/kg bodyweight	79 (55)
CSA/MMF	34 (24)
CSA/MMF/PTCy	16 (11)
Everolimus/MMF/ PTCy	5 (3)
Everolimus/MMF	4 (3)
CSA/MMF/KRP	3 (2)
Other^b^	3 (2)

*AML* acute myeloid leukemia, *MDS* myelodysplastic syndrome, *MPN* myeloproliferative syndrome, *NHL* non-Hodgkin Lymphoma, *ALL* acute lymphoblastic leukemia, *MM* multiple myeloma, *HL* Hodgkin lymphoma, *HCT* hematopoietic stem cell transplantation, *MAC* myeloablative conditioning, *RIC* reduced intensity conditioning, *MRD* matched related donor, *MUD* matched unrelated donor, *MMUD* mismatched unrelated donor, *PBSC* peripheral blood stem cells, *BM* bone marrow, *CSA* cyclosporine, *MMF* mycophenolate mofetil, *ATG* anti thymocyte globulin (Grafalon/formerly ATG-Fresenius), *PTCy* post transplantation cyclophosphamide, *MTX* methotrexate, *KRP* sphigosine-1-phosphate receptor agonist.

^a^Other: acute biphenotypic leukemia (1), severe aplastic anemia (1).

^b^Other: Everolimus/MMF/ATG30mg/kg bodyweight (1), CSA/MTX/KRP (1), MMF/Tacrolimus/Cyclophosphamide (1).

**Table 2:** GVHD characteristics.

Overall aGVHD Grade at onset		Total *n* = 144 (%)
II–IV		136 (94)
III–IV		108 (75)
GI GVHD Clinical Stage	II–IV	105 (73)
	III–IV	85 (59)
Liver GVHD Clinical Stage	II–IV	26 (18)
	III–IV	12 (8)
Skin GVHD Clinical Stage	II–IV	58 (40)
	III–IV	41 (28)
max. GI GVHD	II–IV	142 (99)
	III–IV	97 (67)
cGVHD	Mild	7 (5)
	Moderate	14 (10)
	Severe	42 (29)
	No cGvHD/not known	81 (56)
aGVHD onset		Median (range)
Time to aGvHD onset (days)		27.5 (8–222)
Time to max. GI GvHD onset (days)		37.0 (8–256)
		SLT patients *n* = 77 (%)
GI GVHD at SLT	II–IV	68 (88.3)
	III–IV	47 (61.0)
	n.a.	2 (2.6)

*aGVHD* acute GVHD, *cGVHD* chronic GVHD, *GI GVHD* gastro-intestinal GVHD, *SLT* second-line therapy.

### Responses of lower GI-aGVHD to second-line therapy

**Fig. 1:**
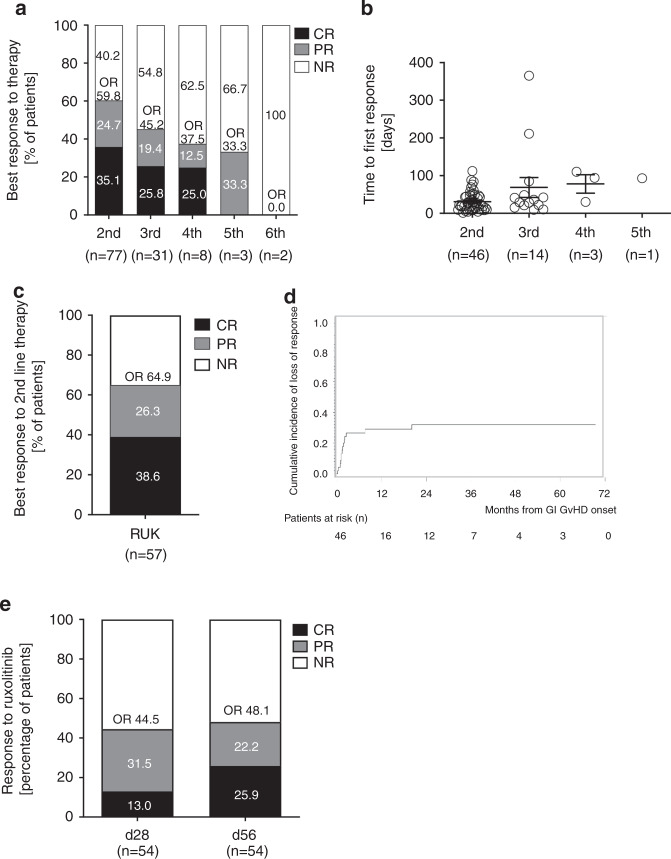
Response of lower GI-aGVHD to subsequent therapy lines. **a** Percentage of patients who achieved partial and complete best response divided by line of therapy in which first response occurred. **b** Time (days) from start of therapy to achievement of first response divided by line of therapy in which first response occurred. **c** Percentage of patients who achieved partial and complete best response during SLT with ruxolitinib. **d** Cumulative incidence of loss of response to SLT, from first response to progression of acute GI GVHD or introduction of additional systemic therapy for acute GI GVHD; censored at last follow-up; competing events were the evolution into chronic GVHD or death without progression of acute GI GVHD. **e** Percentage of patients who achieved partial response or complete response of lower GI-aGVHD at day 28 and day 56 after ruxolitinib. Data on the response at day 28 and 56 was not available for 3 patients, therefore only 54 patients are reported here not 57.

The majority of the patients presented with severe GI-aGVHD at the time of overall GVHD onset. A severe gastrointestinal involvement (grade III–IV GI GVHD) occurred in 59% of patients at GVHD onset and in additional 8.3% of the patients during the disease course. GVHD therapies are presented in Supplementary Table 1. Fifty-seven percent (82/144) of patients were classified as SR-GI-GVHD. Of these, 77 patients received a SLT for GI-GVHD and 5 died before any other systemic treatment could be started. The most common SLT was ruxolitinib, given to 74.0% of the SR-GI-GVHD patients who started a SLT. Other SLT options included extracorporeal photopheresis (6.5%), everolimus (6.5%), cyclosporine (2.6%) and alemtuzumab (1.3%). 9.1% of the SR-GI-GVHD patients received two systemic treatments simultaneously. Considering the best response to therapy, of the 77 patients who received a SLT, 46 (59.8%) achieved a response (PR or CR), of which 27 (35.1%) achieved CRs (Fig. 1a). OR and CR rates decreased with every subsequent therapy-line, starting at 59.8% and 35.1% respectively in the second line and reaching 45.2 and 25.8% in third and 37.5 and 25% in fourth line. Only 33.3% of the patients achieved a PR after the fifth-line and no patient reached a CR after the fifth-line (Fig. 1a). Time to response was within 2–4 weeks in most patients (Fig. 1b). Response to SLT, was observed in patients treated with ruxolitinib with an OR and CR in 64.9 and 38.6%, respectively (Fig. 1c). OR and CR to other agents were 46.2 and 23.1% respectively (data not shown). In the responding patients no difference was observed in terms of time needed to achieve a first response (Supplementary Fig. 1A). Stage III and IV GI-GVHD was observed in 47.4% and 42.1%, respectively in patients who received ruxolitinib, whereas stage III and IV GI-GVHD was observed in 76.9% and 23.1%, respectively in patients receiving agents other than ruxolitinib (Supplementary Fig. 1B). The cumulative incidence of loss of response in all patients who received SLT was 26% at 3 months and reached a plateau at 31.5% by 24 months (Fig. 1d). Outcome at day 28 and 56 after SLT was analyzed. Partial and CR of lower GI-aGVHD to ruxolitinib on day 28 were 31.5% and 13.0%, respectively (Fig. 1e). Partial and CR of lower GI-aGVHD to ruxolitinib on day 56 were 22.2% and 25.9%, respectively (Fig. 1e).

### Survival is higher in patients with glucocorticoid sensitive GI-GVHD

**Fig. 2:**
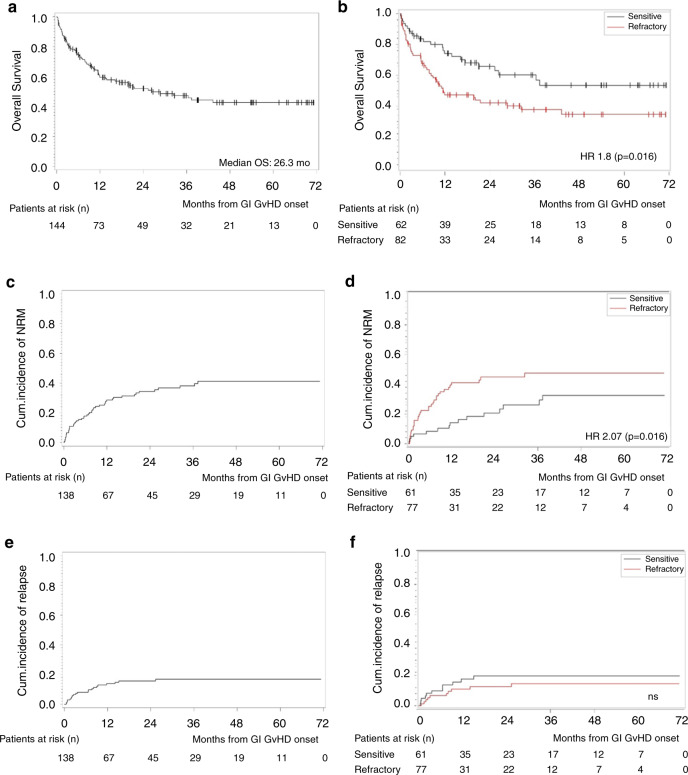
Outcome of glucocorticoid sensitive and glucocorticoid refractory lower GI-aGVHD. **a** Kaplan–Meier estimates for OS probability from GI GVHD onset to death of any cause, censored at last follow-up, for whole cohort of patients. **b** Kaplan–Meier estimates for OS probability from GI GVHD onset to death of any cause, censored at last follow-up, for glucocorticoid sensitive and glucocorticoid refractory patients. Hazard Ratio is calculated with Cox Regression model. **c** Cumulative incidence of non-relapse-related mortality from GI GVHD onset to death not preceded by relapse/progression of primary cancer for whole cohort of patients; censored at last follow-up; competing event being relapse/progression of primary cancer. **d** Cumulative incidence of non-relapse-related mortality from GI GVHD onset to death not preceded by relapse/progression of primary cancer for glucocorticoid sensitive and glucocorticoid refractory patients; censored at last follow-up; competing event being relapse/progression of primary cancer. Hazard Ratio is calculated with Fine-Gray Regression model. **e** Cumulative incidence of relapse/progression of primary cancer from GI GVHD onset for whole cohort of patients; censored at last follow-up; competing event being death before occurrence of relapse/progression of primary cancer. **f** Cumulative incidence of relapse/progression of primary cancer from GI GVHD onset for glucocorticoid sensitive and glucocorticoid refractory patients; censored at last follow-up; competing event being death before occurrence of relapse/progression of primary cancer.

At a median follow-up of 36 months, the median OS in the whole cohort was 26.3 months (95% CI: 13.5 months-not reached) (Fig. 2a). A subgroup of 9 patients developing GI-GVHD after the second allo-HCT experienced a trend toward reduced OS (Supplementary Table 3). Survival was higher in patients with glucocorticoid sensitive GI-GVHD (Fig. 2b). The median OS was not reached in the glucocorticoid sensitive group while it was 11.9 months in the glucocorticoid refractory group (hazard ratio for death 1.8, *p* = 0.016) (Fig. 2b). The cumulative incidence of non-relapse-related mortality at 12 months was 28.6% (Fig. 2c) and the patients with SR-GVHD showed a significantly increased incidence of non-relapse-mortality compared to the glucocorticoid sensitive group (39.2 vs 14.3% at 12 months, *p* = 0.016) (Fig. 2d). The majority of patients that died of non-relapse mortality had an active infection at the time point of death (62.5%), while 2% of the patients died of bleeding. To evaluate the impact of prolonged immunosuppression on the primary malignancy, we analyzed the cumulative incidence of relapse/progression of the primary malignancy after GI-GVHD onset in the two groups of glucocorticoid sensitive or refractory patients and found no difference between the groups (Fig. 2e, f).

### Complete response of lower GI-aGVHD at day 28 correlates with improved outcome

**Fig. 3:**
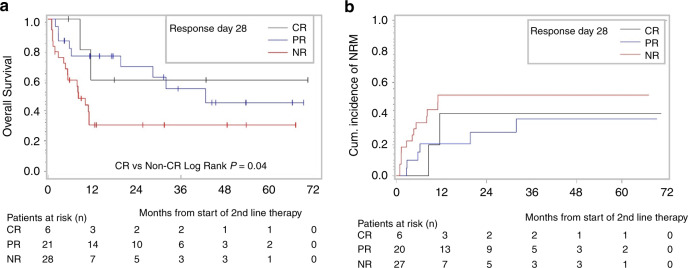
Impact of response to ruxolitinib on survival. **a** Kaplan–Meier estimate for OS probability from begin of ruxolitinib to death of any cause, censored at last follow-up, divided by response at day 28 (CR: complete response, PR: partial response, NR: non-responders). Log-Rank is calculated with landmark analysis, eliminating all patients who did not reach day 28 from the analysis. **b** Cumulative incidence of non-relapse-related mortality from start of ruxolitinib to death not preceded by relapse/progression of primary cancer; censored at last follow-up; competing event being relapse/progression of primary cancer, divided by response at day 28 (CR: complete response, PR: partial response, NR: non-responders). All patients who did not reach day 28 were eliminated from the landmark analysis.

Patients achieved different outcomes when divided according to their response to SLT. Patients that achieved CR at day 28 had a higher OS than patients not achieving a CR (PR or than non-responders): median OS of CR-patients was not reached, median OS was 42.6 and 8.2 months of PR-patients and of non-responders, respectively (CR vs non-CR: *p* = 0.04, Fig. 3a). The unfavorable outcome of patients refractory to SLT is reflected by the highest non-relapse mortality (Fig. 3b).

## Discussion

To describe GI-GVHD outcomes we analyzed data collected over a period of 6 years on patients developing GI-GVHD at MCUF. The motivation was that previous reports on GI-GVHD had analyzed patients in the era before ruxolitinib [29, 30] and that novel therapies that target selectively GI-GVHD showed efficacy in mice. These GI-GVHD regenerative approaches include interleukin-22 (IL-22) [24, 25], R-spondin [26, 27], and GLP-2 [28]. To test these GI regenerative approaches in patients, baseline information on therapy response and survival of GI-GVHD patients is crucial because reports on the cumulative GVHD response will also include skin and liver GVHD responses, which are unlikely impacted by the GI selective regenerative approaches.

Glucocorticoid-resistance in our study population was slightly higher than in previous reports on overall GVHD [12, 17, 32]. This could be related to the exclusion of skin- and liver-only GVHD in our analysis, considering that GI-GVHD has been associated with high risk of glucocorticoid-resistance [17]. In agreement with our data, the majority of patients with acute SR-GVHD enrolled in the REACH2 clinical trial [20] presented with lower GI-tract aGVHD (68.3%). Of note, baseline patients´ characteristics in our centre were similar to the REACH2 clinical trial, especially in terms of donor type (non-related donor for 64.5 and 65.7%; related donor for 35.4 and 34% respectively) and GVHD severity (Grade III–IV acute GVHD was 75% and 64.1%, respectively). Together with a lower overall GVHD grade, the lack of GI involvement was reported in the REACH1 trial as the feature associated with the higher OR to ruxolitinib (ORR 76.2%, CI 52.8–91.8) [33]. Therefore we believe, specific data on GI involvement are fundamental for future trials using ruxolitinib combined with lower GI regenerative therapies.

In our analysis, the rate of acute SR-GI-GVHD was high (57%) which was connected to a high rate of non-relapse-mortality (39.2% at 1 year, 47.4% at 5 years) and low OS (49.1% at 1 years, 34.8% at 5 years). This is consistent with previous reports [6, 34] indicating that our patient cohort was comparable to reported GVHD cohorts. In our cohort best OR to ruxolitinib was 64.9% while OR to SLT was 46.2% and CRs were achieved in 38.6 vs 23.1%, respectively. Recently, a small real-world analysis of 23 patients with acute GVHD treated with ruxolitinib reported similar results as our analysis [35].

Response to ruxolitinib at day 28 was 44.5% in this real-wold GI-aGVHD cohort, indicating the need for new therapies to be combined with ruxolitinib, that act differently e.g., by enhancing intestinal regeneration. The low response rate could be because we analyzed only GI-aGVHD patients only while other reports included all organ aGVHD. For example GI-aGVHD is typically more therapy-resistant than isolated skin GVHD. In addition, response to ruxolitinib that we observed here is still higher compared to the response rate reported in other studies with an OR of 27.4% in SR-GVHD [17]. Response of GI-aGVHD to SLT other than ruxolitinib was comparable to previous reports [20]. The effectiveness of ruxolitinib for SR-GI-GVHD seemed higher than that of other agents, even though in this analysis, due to lack of randomization, the patients who received ruxolitinib presented with a higher GI-GVHD stage and no direct comparison was made.

Few data exist on subsequent therapies after SLT. In our analysis we observed ORs after third- and fourth-line therapy (45.2% and 37.5% respectively). These data suggest that more studies on therapeutic strategies after SLT are needed to improve treatment outcome.

Survival data of our analysis clearly confirm the dismal outcome of patients with SR-GI-GVHD in terms of OS and non-relapse-mortality reported by others, supporting the importance of early and intensive treatment of SR-GI-GVHD patients [11]. Our analysis also shows that SR-GVHD patients can achieve a comparable outcome to glucocorticoid sensitive patients if responding to SLT with a CR indicating that CR must be the therapeutic goal. These data indicate the need for novel strategies such as tissue regenerative approaches in order to improve the outcome of SR-GI-GVHD.

In the context of prolonged immunosuppression after allo-HCT due to GVHD it is important to assess the rate of relapse of primary malignancy. We did not observed an increased incidence of relapse/progression of primary malignancy in the cohort of patients that received a prolonged immunosuppression due to SR-GI-GVHD. This could be due to a strong graft versus tumor effect (GVT) in patients with severe GVHD [36, 37]. In addition previous reports showed that ruxolitinib reduced GVHD [38] but did not interfere with GVT effects in mice [39].

Several biomarkers have been analyzed in the context of acute GVHD to stratify risk and to predict outcome [40–42]. These markers are not routinely tested at our centre, therefore we cannot make any statement on the relevance of biomarkers in our analysis of GI-aGVHD patients.

Our report describes the real-world outcome of GI-GVHD patients treated in the era of ruxolitinib. The retrospective design of this analysis allows only to remain on a descriptive level, but the findings can be useful as a basis for the design of future prospective studies that aim at improving the outcome of patients with SR-GI-GVHD using novel GI-regenerative approaches.

## Supplementary information

Suppl Table 1

Suppl Table 2

Suppl Table 3

Suppl Table 4

Suppl Figures and Tables

## Data Availability

All data are available upon request.
