# Supplementary material for: Therapy response of glucocorticoid-refractory acute GVHD of the lower intestinal tract
Source: Bone Marrow Transplant. 2022 Jun 29;57(10):1500–6. doi: 10.1038/s41409-022-01741-3 (PMC9532244; doi:10.1038/s41409-022-01741-3)
Supplement: Supplementary file 1 — Suppl Table 1 [file 41409_2022_1741_MOESM1_ESM.docx]

**Suppl. Table 1 – GVHD related therapies**

| Therapies | n (%) |
| --- | --- |
| *Died before start 2nd line therapy* | 5 (3.5) |
| 2^nd^ line therapy | 77 (53.5) |
| Ruxolitinib - n (% of 2^nd^) | 57 (74.0) |
| ECP - n (% of 2^nd^) | 5 (6.5) |
| Everolimus - n (% of 2^nd^) | 5 (6.5) |
| CyA - n (% of 2^nd^) | 2 (2.6) |
| Alemtuzumab - n (% of 2^nd^) | 1 (1.3) |
| Combination Therapy* - n (% of 2^nd^) | 7 (9.1) |
| *Died before start 3rd line therapy* - *n (% of 2^nd^)* | 9 (12) |
| 3rd line therapy - n (% of 2^nd^) | 31 (40) |
| Ruxolitinib - n (% of 3^rd^) | 7 (23) |
| ECP - n (% of 3^rd^) | 13 (42) |
| Everolimus - n, (% of 3^rd^) | 6 (19) |
| Alemtuzumab - n (% of 3^rd^) | 3 (10) |
| Cyclophosphamid - n (% of 3^rd^) | 1 (3) |
| Abatacept - n (% of 3rd) | 1 (3) |
| *Died before start 4th line therapy* - *n, (% of 3^rd^)* | 6 (19) |
| 4th line therapy - n (% of 3^rd^) | 8 (26) |
| ECP - n, (% of 4^th^) | 2 (25) |
| CyA - n, (% of 4^th^) | 1 (12.5) |
| Everolimus - n, (% of 4^th^) | 2 (25) |
| MTX - n, (% of 4th) | 3 (37.5) |
| *Died before start 5th line therapy - n, (% of 4^rd^)* | 1 (12.5) |
| 5th line therapy - n, (% of 4^th^) | 3 (37.5) |
| ECP - n, (% of 5^th^) | 1 (33.3) |
| Everolimus - n, (% of 5^th^) | 1 (33.3) |
| MTX - n, (% of 5^th^) | 1 (33.3) |
| 6th line therapy - n, (% of 5^th^) | 2 (67) |
| Alemtuzumab – n, (% of 6^th^) | 2 (100) |
| * Ruxolitinib+ECP (4), Ruxolitinib+CyA (1), ECP+CyA (1), Ruxolitinib+Abatacept (1) | |

Abbreviations: aGvHD: acute GvHD, cGvHD: chronic GvHD, GI GvHD: gastro-intestinal GvHD, ECP: extracorporal photoapherese.
