# Supplementary material for: Therapy response of glucocorticoid-refractory acute GVHD of the lower intestinal tract
Source: Bone Marrow Transplant. 2022 Jun 29;57(10):1500–6. doi: 10.1038/s41409-022-01741-3 (PMC9532244; doi:10.1038/s41409-022-01741-3)
Supplement: Supplementary file 2 — Suppl Table 2 [file 41409_2022_1741_MOESM2_ESM.docx]

**Suppl. Table 2 – Absolute dosage of glucocorticoid-therapy given for aGVHD affecting organs other than the lower GI tract to the 8 patients who later developed GI GVHD.**

| Patient | Treatment (absolute dosage per day) |
| --- | --- |
| 1 | 50mg prednisolon for 5 days, then taper |
| 2 | 50mg prednisolon for 5 days, then taper |
| 3 | 40mg prednisolon for 5 days, then taper |
| 4 | 100mg prednisolon for 2 days, then stop |
| 5 | 180mg prednisolon for 2 days, then taper |
| 6 | 50mg prednisolon for 5 days, then taper |
| 7 | 100mg prednisolon for 5 days, then taper |
| 8 | 20mg prednisolon for 5 days, then taper |
