# Supplementary material for: Therapy response of glucocorticoid-refractory acute GVHD of the lower intestinal tract
Source: Bone Marrow Transplant. 2022 Jun 29;57(10):1500–6. doi: 10.1038/s41409-022-01741-3 (PMC9532244; doi:10.1038/s41409-022-01741-3)
Supplement: Supplementary file 3 — Suppl Table 3 [file 41409_2022_1741_MOESM3_ESM.docx]

**Suppl. Table 3 – outcomes of 1^st^ vs. 2^nd^ HCT**

|  | Whole cohort  (n=144) | 1^st^ HCT  (n=135) | 2^nd^ HCT  (n=9) |
| --- | --- | --- | --- |
| Median OS (months) | 26.3 | 36.4 | 11.9 |
| Log Rank (Mantel Cox) Test: 3.838 (p=0.0501) | | | |

Abbreviations: OS: overall survival, HCT: hematopoietic cell transplantation.
