# Supplementary material for: Therapy response of glucocorticoid-refractory acute GVHD of the lower intestinal tract
Source: Bone Marrow Transplant. 2022 Jun 29;57(10):1500–6. doi: 10.1038/s41409-022-01741-3 (PMC9532244; doi:10.1038/s41409-022-01741-3)
Supplement: Supplementary file 4 — Suppl Table 4 [file 41409_2022_1741_MOESM4_ESM.docx]

**Suppl. Table 4 – Steroid-refractory criteria and steroid dose**

| **SR GI GVHD, n (%)** | **n=82 (%)** |
| --- | --- |
| Steroid refractory |  |
| Progression after at least 3 days | 29 (37.7) |
| No improvement after 7 days | 37 (48.1) |
| Steroid dependent |  |
| GVHD flare during steroid taper | 14 (18.2) |
| Data not available | 2 (2.6) |

|  | **Steroid dose at GVHD onset (n=82)** | **Steroid dose at SLT**  **(n=77)** |
| --- | --- | --- |
| median dose mg/kg (range) | 1.7 (0.5-3.3) | 1.7 (0.1-3.0) |
|  | SR patients, n (%) | SLT patients, n (%) |
| 0.5-1 mg/kg | 14 (17.1) | 15 (19.5) |
| 1-2 mg/kg | 31 (37.8) | 31 (40.3) |
| ≥2 mg/kg | 35 (42.7) | 29 (37.7) |
| Data not available | 2 (2.4) | 2 (2.6) |

Abbreviations: SR GI GVHD: steroid-refractory gastrointestinal GVHD, SLT: second-line therapy.
