# Supplementary material for: Therapy response of glucocorticoid-refractory acute GVHD of the lower intestinal tract
Source: Bone Marrow Transplant. 2022 Jun 29;57(10):1500–6. doi: 10.1038/s41409-022-01741-3 (PMC9532244; doi:10.1038/s41409-022-01741-3)
Supplement: Supplementary file 5 — Suppl Figures and Tables [file 41409_2022_1741_MOESM5_ESM.pdf]

Suppl. Figure 1

a

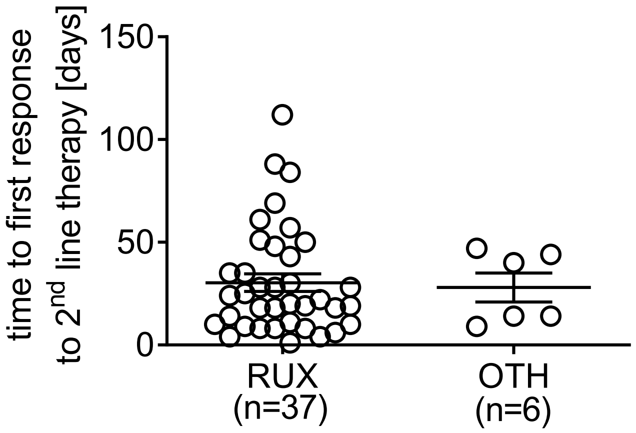

b

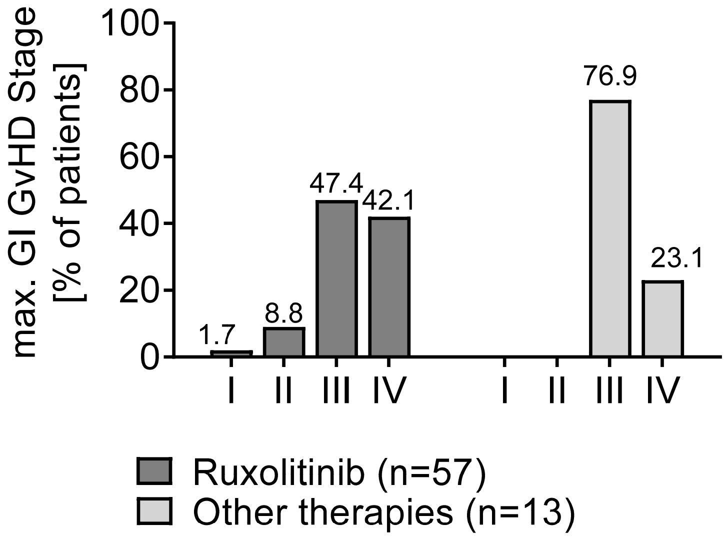

Suppl. Figure 1

A) Time (days) from start of SLT to achievement of first response divided by therapy-agent used (either ruxolitinib or another single agent).

B) Percentage of patients with GI GVHD stage III or IV divided by therapy-agent used as SLT (either ruxolitinib or another single agent).

Suppl. Table 1 – GVHD related therapies

**Therapies**	**n (%)**
Died before start 2nd line therapy	5 (3.5)
2nd line therapy	77 (53.5)
Ruxolitinib - n (% of 2nd)	57 (74.0)
ECP - n (% of 2nd)	5 (6.5)
Everolimus - n (% of 2nd)	5 (6.5)
CyA - n (% of 2nd)	2 (2.6)
Alemtuzumab - n (% of 2nd)	1 (1.3)
Combination Therapy* - n (% of 2nd)	7 (9.1)
Died before start 3rd line therapy - n (% of 2nd)	9 (12)
3rd line therapy - n (% of 2nd)	31 (40)
Ruxolitinib - n (% of 3rd)	7 (23)
ECP - n (% of 3rd)	13 (42)
Everolimus - n, (% of 3rd)	6 (19)
Alemtuzumab - n (% of 3rd)	3 (10)
Cyclophosphamid - n (% of 3rd)	1 (3)
Abatacept - n (% of 3rd)	1 (3)
Died before start 4th line therapy - n, (% of 3rd)	6 (19)
4th line therapy - n (% of 3rd)	8 (26)
ECP - n, (% of 4th)	2 (25)
CyA - n, (% of 4th)	1 (12.5)
Everolimus - n, (% of 4th)	2 (25)
MTX - n, (% of 4th)	3 (37.5)
Died before start 5th line therapy - n, (% of 4rd)	1 (12.5)
5th line therapy - n, (% of 4th)	3 (37.5)
ECP - n, (% of 5th)	1 (33.3)
Everolimus - n, (% of 5th)	1 (33.3)
MTX - n, (% of 5th)	1 (33.3)
6th line therapy - n, (% of 5th)	2 (67)
Alemtuzumab – n, (% of 6th)	2 (100)

* Ruxolitinib+ECP (4), Ruxolitinib+CyA (1), ECP+CyA (1), Ruxolitinib+Abatacept (1)

Abbreviations: aGvHD: acute GvHD, cGvHD: chronic GvHD, GI GvHD: gastro-intestinal GvHD, ECP: extracorporal photoapherese.

Suppl. Table 2 – Absolute dosage of glucocorticosteroid-therapy given for aGVHD affecting organs other than the lower GI tract to the 8 patients who later developed GI GVHD.

Patient	Treatment (absolute dosage per day)
1	50mg prednisolon for 5 days, then taper
2	50mg prednisolon for 5 days, then taper
3	40mg prednisolon for 5 days, then taper
4	100mg prednisolon for 2 days, then stop
5	180mg prednisolon for 2 days, then taper
6	50mg prednisolon for 5 days, then taper
7	100mg prednisolon for 5 days, then taper
8	20mg prednisolon for 5 days, then taper

Suppl. Table 3 – outcomes of 1st vs. 2nd HCT

	Whole cohort	1st HCT	2nd HCT
Median OS (months)	26.3	36.4	11.9
Log Rank (Mantel Cox) Test: 3.838 (p=0.0501)			

Abbreviations: OS: overall survival, HCT: hematopoietic cell transplantation.

Suppl. Table 4 – Steroid-refractory criteria and steroid dose

**SR GI GVHD**	**n=82 (%)**
Steroid refractory	
Progression after at least 3 days	29 (37.7)
No improvement after 7 days	37 (48.1)
Steroid dependent	
GVHD flare during steroid taper	14 (18.2)
Data not available	2 (2.6)

	**Steroid dose at GVHD onset**	**Steroid dose at SLT**
median dose (range)	1.7 (0.5-3.3)	1.7 (0.1-3.0)
	SR patients n=82 (%)	SLT patients n=77 (%)
0.5-1 mg/kg	14 (17.1)	15 (19.5)
1-2 mg/kg	31 (37.8)	31 (40.3)
≥2 mg/kg	35 (42.7)	29 (37.7)
Data not available	2 (2.4)	2 (2.6)

Abbreviations: SR GI GVHD: steroid-refractory gastrointestinal GVHD, SLT: second-line therapy.
